# Mammalian chromosome–telomere length dynamics

**DOI:** 10.1098/rsos.180492

**Published:** 2018-07-25

**Authors:** Amy R. Klegarth, Dan T. A. Eisenberg

**Affiliations:** 1 Department of Anthropology, University of Washington, 314 Denny Hall, Seattle, WA 98105, USA; 2 Center for Studies in Demography and Ecology, University of Washington, 230 Raitt Hall, Seattle, WA 98105, USA

**Keywords:** telomere length, mammals, chromosome size, genome size, *C*-value, evolutionary lag

## Abstract

Individual chromosome arms have specific individual telomere lengths (TLs). Past studies within species have shown strong positive correlations between individual chromosome length and TL at that chromosome. While the reasons for these associations are unclear, the strength and consistency of the associations across disparate taxa suggest that this is important to telomere biology and should be explored further. If TL is primarily determined by chromosome length, then chromosome length should be considered and controlled for in cross-species analyses of TL. Here, we employ a cross-species approach to explore whether the chromosome length–TL association observed intraspecifically is a determinant of mean TL across species. Data were compiled from two studies characterizing TL across a range of mammalian taxa and analysed in a phylogenetic framework. We found no significant relationship between TL and chromosome size across mammals or within mammalians orders. The pattern trends in the expected direction and we suggest may be masked by evolutionary lag effects.

## 1. Background

Telomeres are a repetitive nucleotide sequence that cap the ends of linear chromosomes in eukaryotic organisms and protect chromosome ends from degradation due to incomplete end replication [1]. Research on telomeres often focuses on telomere length (TL) because shortened and shortening of telomeres have been linked to health and ageing indicators [2]. Owing to logistical constraints, only mean TL is typically measured, despite the fact that every individual chromosome end has a characteristic TL [3–5]. The fine-scale specificity of TL at the level of chromosome arms suggests a great deal of telomere biology and patterns of variation in TL could be masked by only considering mean TL. Hinting at important TL dynamics, several human studies from independent laboratories have observed a consistent, positive linear relationship (*r* = 0.6–0.76) between whole chromosome size and TL on individual chromosomes [6–8]. Similar associations exist between TL and individual chromosome arm length across chromosomes (*r* = 0.57–0.79) [3,9,10].

While most extensively studied in humans, a positive relationship between chromosome size and TL has also been noted within several other species. In mammals, this association is found in three species of laboratory mice [11] as well as in Chinese hamsters [12]. The same relationship has also been noted among angiosperm plants [13,14], grasses from the genus *Pennisetum* [15], yeast [16–18] and protozoans [19]. Given that this pattern has been observed across such a broad distribution of taxa, the relationship may be a highly conserved aspect of telomere biology. However, an exception to this general pattern does appear in birds and reptiles, where microchromosomes sometimes possess mega-telomeres [20,21]. This is likely due to the high recombination rates observed among microchromosomes [22].

Despite the reported associations between TL and chromosome size across several disparate taxa, it remains unclear why this pattern exists. Studies focused on chromosome-specific TL have identified subtelomeric single nucleotide polymorphisms associated with TL that may be indicative of local genetic effects contributing to TL at specific chromosomes and chromosome arms [3,7,8,16–19]. Experiments on yeast have revealed an antagonism between telomeres and centromeres [23], which Slijepcevic [24] proposes as a possible mechanism to explain the correlation between TL and individual chromosome arm lengths. This telomere–centromere antagonism likely occurs during meiosis when telomeres and centromeres are pulled in opposite directions. The telomere–centromere antagonism suggests that close proximity of telomeres to centromeres can lead to instability, ultimately resulting in a need for epigenetic suppression of TL on the shorter, p-arms of chromosomes [24].

Across species, mean chromosome length varies considerably [25,26]. Work on the evolutionary determinants and biology of TL across species has predominantly considered species' mean TL [27–31]. If TL is determined by chromosome size or arm length, then the pattern of division of genomes of varying size into differing numbers of chromosomes is expected to result in differing mean TL [32]. For example, given two hypothetical species, both with a genome size of 5 gigabases, but one with five chromosomes (average chromosome size of 1 gigabase) and another with 50 chromosomes (average chromosome size of 0.1 gigabases), we would expect the species with shorter chromosomes to have much shorter mean TL.

In this study, we aim to test whether a continuous relationship exists between mean chromosome size and mean TL across mammalian species. Given the disparate taxa within which the relationship between mean TL and chromosome size has already been observed (humans and multiple rodent species) we expect to uncover the same pattern across mammals broadly. Alternatively, it is possible that the relationship between chromosome size and TL only exists in specific subsets of mammalian taxa such as primates and rodents. If a continuous relationship exists between TL and chromosome size across mammals or even within specific subsets of mammalian taxa it suggests the pattern has been conserved and may be an important, if overlooked, an aspect of functional telomere biology.

## 2. Material and methods

*Dataset:* Individual taxa of interest (*N* = 45 species) were curated from two studies measuring TL across a range of mammals, with a subset of five species overlapping between studies [29,31]. In the five species which overlapped between datasets a 1 kb longer TL in the Gomes *et al.* dataset predicted a 1.46 kb longer TL (95% CI: +0.72 to + 2.21) in the Seluanov dataset with a *y*-intercept of 4.41 (95% CI: −21.52 to + 30.34). *C*-values, or picograms of DNA per haploid nucleus, are a measure of genome size and were compiled along with haploid chromosome number using the Animal Genome Size Database (http://www.genomesize.com/) [33] unless otherwise noted (see electronic supplementary material, table S1). These *C*-values estimated via traditional cytogenetic methods strongly correlate to genome sizes measured via sequencing and assembly [34]. Owing to the systemic underestimation of larger genomes when measured via sequencing [34] as well as the limited number of available sequenced mammalian genomes with corresponding TL measures, *C*-values were deemed the most appropriate metric for estimating mean chromosome size. Mean chromosome size was calculated by dividing *C*-values by the haploid number of chromosomes for each queried species and then multiplying by 978 to convert picograms of DNA to megabase pairs (Mbp) of DNA (electronic supplementary material, table S1). TL data were measured via telomere restriction fragment (TRF) analysis for both studies but with some variations in the TRF assay between the two studies (e.g. different restriction enzymes). TL data were from cultured fibroblasts in Gomes *et al*. [31], whereas data from Seluanov *et al*. [29] were from liver DNA. Owing to these differences in cell types and measurement technique between these two studies, we opted to conduct our analysis separately in each TL dataset.

### 2.1. Phylogenetic regression analyses

#### 2.1.1. Tree trimming

A mammalian tree was downloaded [35] and pruned using the *ape* package *droptip* command [36] in R v. 3.3.2 [37]. Separate trees were generated that included all species from Gomes *et al*. [31] (*N* = 39; electronic supplementary material, figure S1) and Seluanov *et al*. [29] (*N* = 11; electronic supplementary material, figure S2) for which data on genome size were available. Separate trees were generated for all orders for which at least seven species were present within the full mammalian phylogeny (electronic supplementary material, figures S3A–S3C). Based on these criteria, two additional trees were generated from the Gomes *et al*. [31] dataset for the orders Primates and Cetartiodactyla (two-toed ungulates). The Seluanov *et al*. [29] dataset is comprised completely of taxa from the order Rodentia (rodents) and served as the third tree for intra-order analyses. All phylogenetic trees were visualized using the Interactive Tree of Life v. 2 (iTOL v. 2) [38].

#### 2.2.2. Analysis of telomere lengths and chromosome size

The relationship between mean chromosome size and TL was tested within a phylogenetic generalized least squares (PGLS) framework with the program BayesTraits v. 2 [39]. Lambda (*λ*), the measure of phylogenetic signal, was estimated for each of the four analyses across 1000 maximum-likelihood trees. Prior to running PGLS models *z*-scores were calculated for each species for both mean chromosome length and TL [40] to test for outliers. Samples were considered an outlier if their *z*-score was greater than 2.50 or less than −2.50. The Indian muntjac, *Muntiacus muntjak*, was the only outlier within the dataset, with an extremely large mean chromosome size. Analyses were subsequently run with and without the Indian muntjac. *p*-values were calculated with a *t*-distribution by first calculating *t*-ratio values from PGLS run *β*s and *β* standard errors. Power analyses were performed using the *pwr.r.test* function from the *pwr* package in R v. 3.3.2 [37] using *r* values calculated in PGLS runs.

## 3. Results

**Figure 1.**
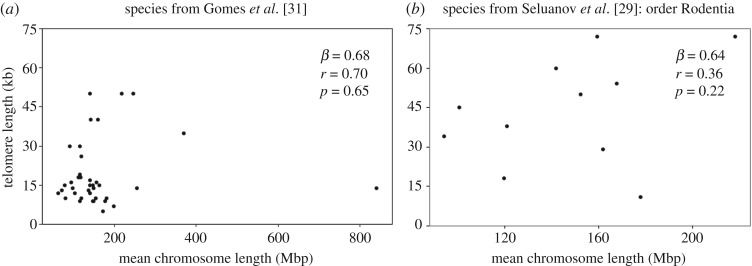
Scatterplots of mean chromosome length (Mbp) and TL (kb) with PGLS-adjusted *β*, *r* and *p*-values for (*a*) Gomes *et al*. dataset, including outliers and (*b*) Seluanov *et al*. dataset. Scatterplots are of raw data and do not reflect phylogenetic adjustment. When the outlier is removed from the Gomes *et al*. dataset, PGLS-adjusted values are as follows: *β* = 0.98, *r* = 0.22, and *p* = 0.16.

No phylogenetic signal for mean chromosome size was detected within orders (*λ* = 0.0000001), but a strong phylogenetic signal is observed across all analysed mammalian taxa once the outlier, *Muntiacus muntjak*, was removed (*λ* = 0.79; table 1). PGLS maximum-likelihood model results for mean chromosome size and TL across mammal species yielded effects in the expected direction but no significant relationships (*p* > 0.05; figure 1*a*,*b*) for either dataset [29,31]. However, when the outlier was removed, a more positive and closer to significant relationship was revealed. All PGLS *β*s were positive (not significant) with the exception of those estimated for the orders Primates and Cetartiodactyla, which had non-significant negative correlations. Our mean power to detect significant (*p* < 0.05) relationships across all six PGLS runs was 18.3%, with the lowest power occurring for the full Gomes *et al*. [31] dataset without the outlier removed (7%). With a sample size of 39, we had 80% power to detect a correlation (*r*) of 0.43.

**Table 1.** Association of mean chromosome size with mean TL from PGLS models.

species list	*N*	*λ*	*β*	*r*	*p*	power (%)
Gomes *et al.* [31]	39	0.00	0.68	0.07	0.65	7
Gomes *et al.* [31]^a^	38	0.79	0.98	0.22	0.16	27
Seluanov *et al.* [29]/order Rodentia	11	0.00	0.64	0.36	0.22	20
order Primates	7	0.00	−3.55	−0.56	0.11	27
order Cetartiodactyla	7	0.00	−6.49	−0.31	0.38	11

^a^PGLS results for Gomes *et al*. [31] dataset with the outlier, *Muntiacus muntjak*, removed.

## 4. Discussion

Across mammalian species, there is a positive, but non-significant, relationship between mean chromosome size and mean TL (figure 1*a*,*b*). Our study had the statistical power to detect a moderate effect size, suggesting that no moderate or larger effect size exists within the currently available data. The removal of the Indian muntjac from the largest dataset increased the magnitude of the positive relationship and decreased the *p*-value from 0.65 to 0.16 (table 1; figure 1*a*). Both the large impact of this single outlier and the overall low power to detect a significant relationship (7–27% across analyses) suggest that sample size is a primary factor contributing to these null results. As new data on TL and genome size for additional mammalian species become available in the coming years, it will become possible to discern the nature of these associations with more certainty (table 1). Our results stand in contrast to many intraspecific studies which have documented significant positive correlations between chromosome size and TL. This contradiction between the intra- versus interspecific patterns suggests that longer telomeres may be an important adaptive response to genomic rearrangements resulting in larger mean chromosome size within the genome of a given species, but that the impact of this relationship dissipates at an interspecific level as other adaptive forces come to play a larger role [41].

The expected relationship of longer telomeres occurring alongside greater mean chromosome size was also not observed interspecifically within the orders Rodentia or Primates, where the strongest evidence for correlations between these traits exists at the intraspecific level [3,6–12]. Neither did the relationship exist within the third order analysed, Cetartiodactyla. Within both the orders Primates and Cetartiodactyla, we report non-significant negative correlations (*r* = −0.56 and *r* = −0.31), suggesting a possible trend opposite to the expected direction. This is particularly notable given these coefficients are inverted when species from the order Primates and Cetartiodactyla are analysed within the context of mammals more broadly (*r* = 0.22). This relationship may be particularly interesting to explore among a larger number of species within both orders in the future.

Large changes in mean chromosome size occur via chromosomal rearrangements that include deletions, duplications and fissions or fusions via inversions or translocations [42]. When major rearrangements occur and functional genes are largely left intact, it can lead to rapid speciation [43,44]. Such karyotypic incompatibility can lead to speciation via breeding isolation, resulting in stronger selection on the two new species to diversify and successfully establish their own niches. It is possible that selection for corresponding TL to new chromosome sizes is not as strong, ultimately resulting in an evolutionary lag between the two traits. A force such as evolutionary lag could account for the observed null results. Unfortunately, large trees with at least 100+ species pairs that share a direct common ancestor are required to be able to reliably detect such a lag [45].

The outlier, the Indian muntjac, provides intriguing anecdotal evidence for a possible evolutionary lag between TL and mean chromosome sizes. Assuming a positive association between TL and chromosome size, the Indian muntjac has much shorter TL than expected given its large chromosome size (figure 1*a*). The Indian muntjac also has the fewest chromosomes of any mammal (2*n* = 6/7) and dramatically differs in karyotype from its close relative the Chinese muntjac (2*n* = 46). These two species are only separated by seven million years of evolution. Indeed, genus *Muntiacus* exhibits some of the fastest rates of change in chromosome number among vertebrates [46]. Rapid reductions in chromosome count within *Muntiacus* are the result of multiple whole chromosome fusions. Initially, such fusions would result in chromosomes capped with the shorter telomeres of the component smaller chromosomes. Another case suggestive of evolutionary lag between chromosome size and TL is found in human chromosome 2. Chromosome 2 resulted from the fusion of two separate chromosomes 4–8 million years ago [47–50]. Consistent with an evolutionary lag, human chromosome 2 possesses shorter relative TL compared to all other human chromosomes relative to individual chromosome size [2].

**Figure 2.**
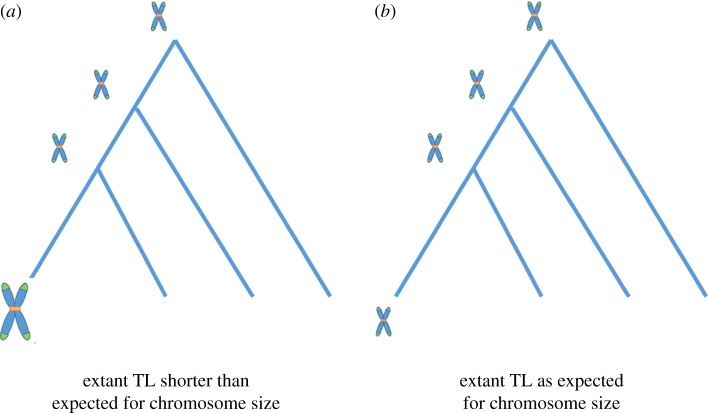
Diagram of expected TL based on chromosome size evolution based on (*a*) a recent fusion creating large chromosomes and (*b*) no recent fissions or fusions.

Unfortunately, little data exist on specific TL at individual chromosomes outside of humans and a select few other species. If chromosome-specific TL were available across a wide range of species, we could empirically test whether chromosomes that result from recent fissions or fusions have respectively longer or shorter telomeres based on expectations. For instance, when longer chromosomes are generated from the fusion of shorter chromosomes, we would expect the new, longer chromosomes to have disproportionately short TL initially (figure 2*a*). By contrast, if no recent change in chromosome size has occurred, enough time may have passed to counteract the effect of evolutionary lag (figure 2*b*). Alternatively, if shorter chromosomes result from the fission of larger chromosomes, we would expect that new shorter chromosomes to initially have disproportionately long TL.

In addition to the proposed evolutionary mechanisms that may obscure the relationship between chromosome size and TL, we must also note some additional methodological limitations. The limited data available on TL for specific chromosomes and chromosome arms across mammalian taxa may mask a stronger relationship between chromosome size and TL. Moreover, the largest datasets on TL in mammals [30,31] both used TRF, a method which cannot perfectly disentangle terminal TL (or functional telomeres) from degenerate or interstitial telomeric sequences. Future multi-species studies should consider employing more precise methodologies such as Q-FISH paired with confocal microscopy [51] to better characterize chromosome arm specific TLs. Associations between chromosome-specific TL and chromosome size may also be impacted by telomere and chromosome positional effects within the nucleus [52].

Here, we demonstrate that the previously observed relationship between total chromosome size or chromosome arm length and TL found within species cannot yet be detected across a varied, but limited range of mammalian species. Sample size limitations and disparities in TL measurements between studies and quantification methods [53,54] underscore the need for more cross-species analyses to occur within individual laboratories using well-calibrated and standardized methodologies for TL quantification. Larger, well-calibrated, multi-species datasets will be necessary to further explore these intrinsic properties of TL evolution to further inform basic TL biology. Moreover, as molecular tools are continually refined, chromosome-specific analyses will become available for a wider breadth of species and enable the examination of these dynamics at much finer phylogenetic scales.

## Supplementary Material

Figure S1

## Supplementary Material

Figure S2

## Supplementary Material

Figure S3

## Supplementary Material

Figure S4

## Supplementary Material

Table S1. Data summary table with outlier test z-scores

## Data accessibility

Data used in this study were compiled from existing databases [33] and publications [29,31,35] unaffiliated with either author or institution. The compiled data are summarized in electronic supplementary material, table S1 and figure S1. No code was developed for this study. All commands used are detailed in the Material and methods of this paper and were performed according to applicable manuals [36,37,39].
