# Supplementary figures and images for: Mammalian chromosome–telomere length dynamics
Source: R Soc Open Sci. 2018 Jul 25;5(7):180492. doi: 10.1098/rsos.180492 (PMC6083700; doi:10.1098/rsos.180492)

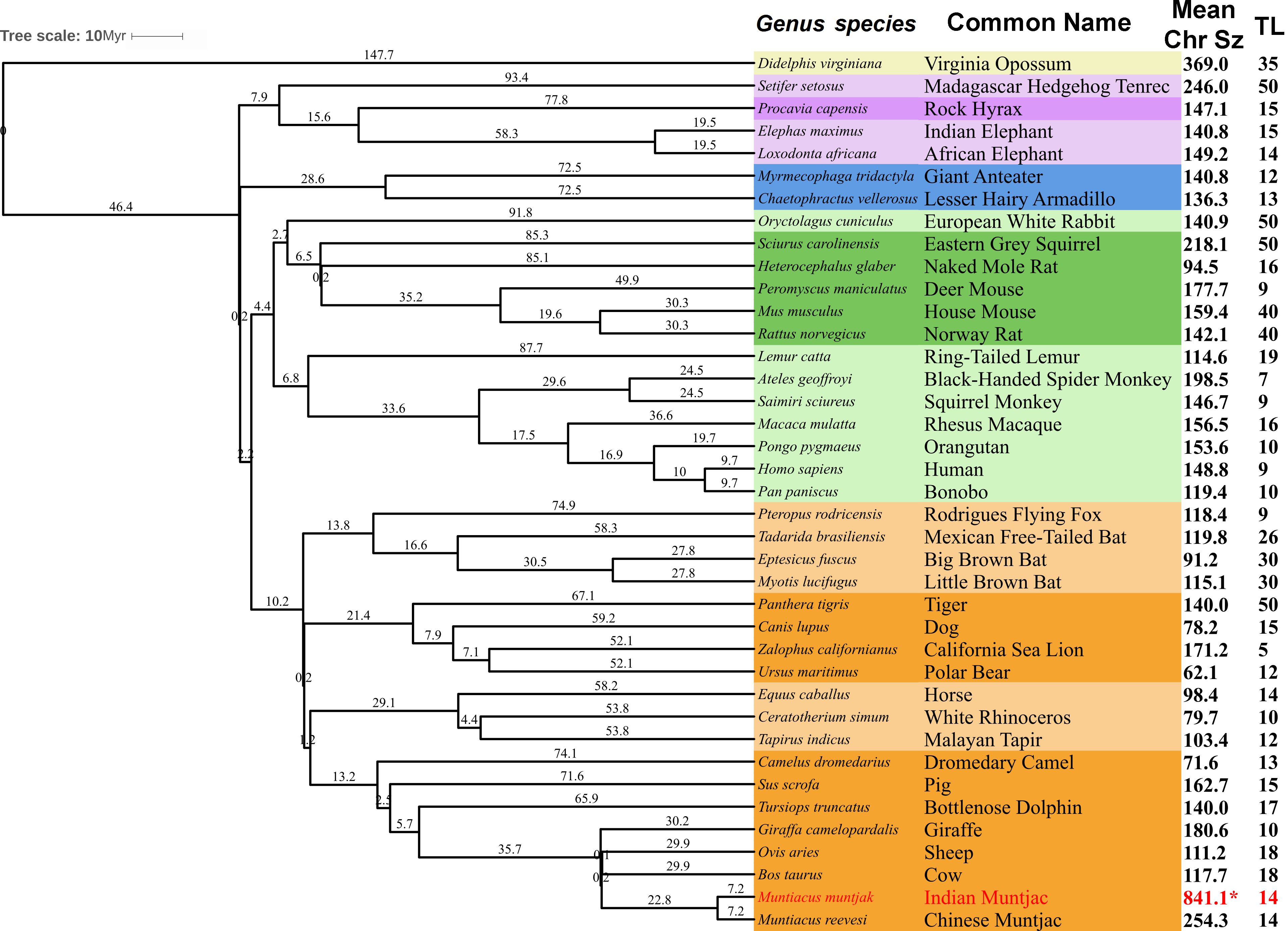

Supplement: Figure S1 [file rsos180492supp1.tif]

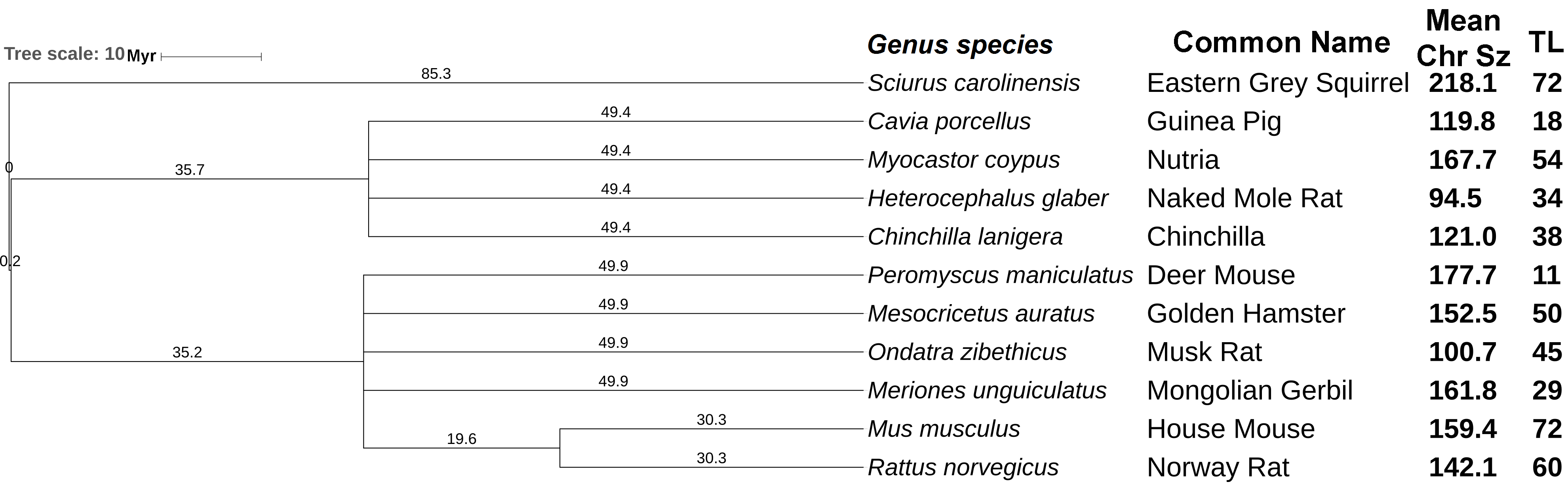

Supplement: Figure S2 [file rsos180492supp2.tif]

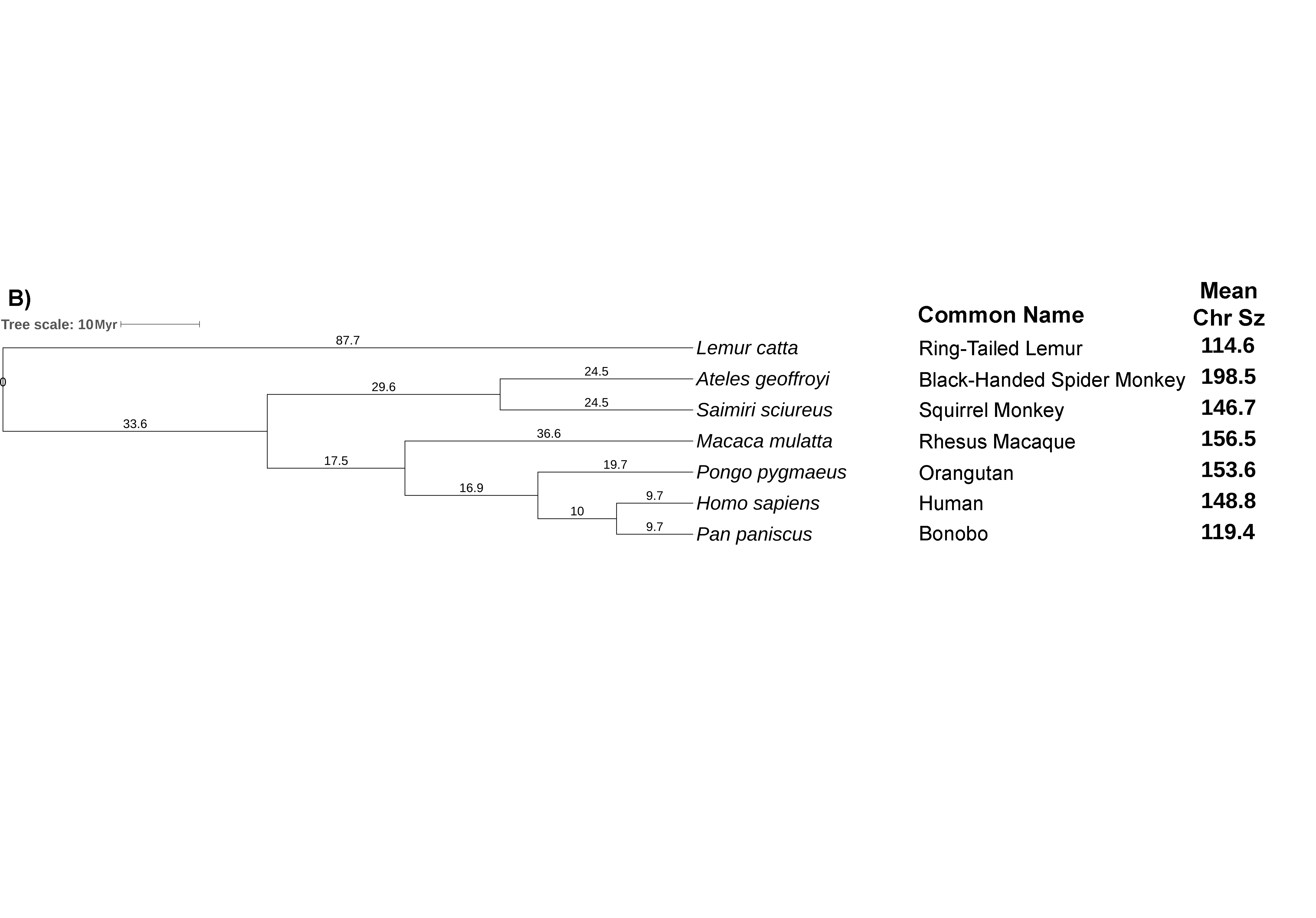

Supplement: Figure S3 [file rsos180492supp3.tif]

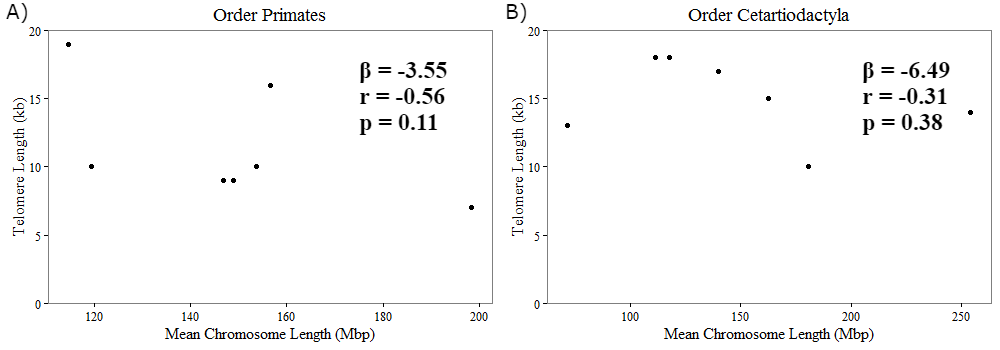

Supplement: Figure S4 [file rsos180492supp4.tiff]
