# Supplementary material for: Mammalian chromosome–telomere length dynamics
Source: R Soc Open Sci. 2018 Jul 25;5(7):180492. doi: 10.1098/rsos.180492 (PMC6083700; doi:10.1098/rsos.180492)
Supplement: Table S1. Data summary table with outlier test z-scores [file rsos180492supp5.docx]

**Table S1. Data summary table with outlier test z-scores**

| **Common Name** | ***Genus species*** | **C-value** | **N Haploid Chromosomes** | **Mean Chromosome Length (Mbp)** | **Mean Chromosome Length z-score** | **Mean TL (kb)** | **Mean TL z-score** |
| --- | --- | --- | --- | --- | --- | --- | --- |
| *Species from Gomes et al. (29)* | | | | | | | |
| African Elephant | *Loxodonta africana* | 4.27 | 28 | 149.1 | -0.11 | 14 | -0.44 |
| Big Brown Bat | *Eptesicus fuscus* | 2.33 | 25 | 91.1 | -0.58 | 30 | 0.78 |
| Black-Handed Spider Monkey | *Ateles geoffroyi* | 3.45 | 17 | 198.5 | 0.29 | 7 | -0.97 |
| Bonobo | *Pan paniscus* | 2.93 | 24 | 119.4 | -0.35 | 10 | -0.74 |
| Bottle-Nose Dolphin | *Tursiops truncatus* | 3.15 | 22 | 140.0 | -0.19 | 17 | -0.21 |
| Camel | *Camelus dromedarius* | 2.71 | 37 | 71.6 | -0.74 | 13 | -0.52 |
| Chinese Muntjac | *Muntiacus reevesi* | 5.98 | 23 | 254.3 | 0.74 | 14 | -0.44 |
| Cow | *Bos taurus* | 3.61 | 30 | 117.7 | -0.37 | 18 | -0.13 |
| Deer Mouse | *Peromyscus maniculatus* | 4.36 | 24 | 177.7 | 0.12 | 9 | -0.82 |
| Dog | *Canis lupus* | 3.12 | 39 | 78.2 | -0.69 | 15 | -0.36 |
| Eastern Grey Squirrel | *Sciurus carolinensis* | 4.46 | 20 | 218.1 | 0.45 | 50 | 2.30 |
| European White Rabbit | *Oryctolagus cuniculus* | 3.17 | 22 | 140.9 | -0.18 | 50 | 2.30 |
| Giant Anteater | *Myrmecophaga tridactyla* | 4.32 | 30 | 140.8 | -0.18 | 12 | -0.59 |
| Giraffe | *Giraffa camelopardalis* | 2.77 | 15 | 180.6 | 0.14 | 10 | -0.74 |
| Hairy Armadillo | *Chaetophractus vellerosus* | 4.18 | 30 | 136.3 | -0.22 | 13 | -0.52 |
| Horse | *Equus caballus* | 3.22 | 32 | 98.4 | -0.52 | 14 | -0.44 |
| House Mouse | *Mus musculus* | 3.26 | 20 | 159.4 | -0.03 | 40 | 1.54 |
| Human | *Homo sapiens* | 3.5 | 23 | 148.8 | -0.11 | 9 | -0.82 |
| Indian Elephant | *Elephas maximus* | 4.03 | 28 | 140.8 | -0.18 | 15 | -0.36 |
| Indian Muntjac | *Muntiacus muntjak* | 2.58 | 3 | 841.1 | **5.50** | 14 | -0.44 |
| Little Brown Bat | *Myotis lucifugus* | 2.59 | 22 | 115.1 | -0.39 | 30 | 0.78 |
| Malaysian Tapir | *Tapirus indicus* | 2.75 | 26 | 103.4 | -0.48 | 12 | -0.59 |
| Mexican Free-Tailed Bat | *Tadarida brasiliensis* | 2.94 | 24 | 119.8 | -0.35 | 26 | 0.47 |
| Naked Mole Rat | *Heterocephalus glaber* | 2.9 | 30 | 94.5 | -0.56 | 16 | -0.29 |
| Norway Rat | *Rattus norvegicus* | 3.05 | 21 | 142.0 | -0.17 | 40 | 1.54 |
| Orangutan | *Pongo pygmaeus* | 3.77 | 24 | 153.6 | -0.08 | 10 | -0.74 |
| Pig | *Sus scrofa* | 3.16 | 19 | 162.7 | 0.00 | 15 | -0.36 |
| Polar Bear | *Ursus maritimus* | 2.35 | 37 | 62.1 | -0.82 | 12 | -0.59 |
| Rhesus Macaque | *Macaca mulatta* | 3.36 | 21 | 156.5 | -0.05 | 16 | -0.29 |
| Ring-Tailed Lemur | *Lemur catta* | 3.28 | 28 | 114.6 | -0.39 | 19 | -0.06 |
| Rock Hyrax | *Procavia capensis* | 4.06 | 27 | 147.1 | -0.13 | 15 | -0.36 |
| Rodrigues' Flying Fox | *Pteropus rodricensis* | 2.23 | 19 | 114.8 | -0.36 | 9 | -0.82 |
| Sea Lion | *Zalophus californianus* | 3.15 | 18 | 171.2 | 0.07 | 5 | -1.13 |
| Sheep | *Ovis aries* | 3.07 | 27 | 111.2 | -0.42 | 18 | -0.13 |
| Squirrel Monkey | *Saimiri sciureus* | 3.3 | 22 | 146.7 | -0.13 | 9 | -0.82 |
| Tiger | *Panthera tigris* | 2.72 | 19 | 140.0 | -0.19 | 50 | 2.30 |
| Virginia Opossum | *Didelphis virginiana* | 4.15 | 11 | 369.0 | 1.67 | 35 | 1.16 |
| White Rhinoceros | *Ceratotherium simum* | 3.34 | 41 | 79.7 | -0.68 | 10 | -0.74 |
| *Species from Seluanov et al. (27)* | | | | | | | |
| Chinchilla | *Chinchilla lanigera* | 3.96 | 32 | 121.0 | -0.75 | 38 | -1.34 |
| Deer Mouse | *Peromyscus maniculatus* | 4.36 | 24 | 177.7 | 0.89 | 11 | -1.71 |
| Eastern Grey Squirrel | *Sciurus carolinensis* | 4.46 | 20 | 218.1 | 2.06 | 72 | 1.46 |
| Golden Hamster | *Mesocricetus auratus* | 3.43 | 22 | 152.5 | 0.16 | 50 | 0.32 |
| Guinea Pig | *Cavia porcellus* | 3.92 | 32 | 119.8 | -0.78 | 18 | -1.34 |
| House Mouse | *Mus musculus* | 3.26 | 20 | 159.4 | 0.36 | 72 | 1.46 |
| Mongolian Gerbil | *Meriones unguiculatus* | 3.64 | 22 | 161.8 | 0.43 | 29 | -0.77 |
| Musk Rat | *Ondatra zibethicus* | 2.78 | 27 | 100.7 | -1.33 | 45 | 0.06 |
| Naked Mole Rat | *Heterocephalus glaber* | 2.9 | 30 | 94.5 | -1.51 | 34 | -0.51 |
| Norway Rat | *Rattus norvegicus* | 3.05 | 21 | 142.0 | -0.14 | 60 | 0.83 |
| Nutria | *Myocastor coypus* | 3.6 | 21 | 167.7 | 0.60 | 54 | 0.52 |

Bolded z-scores indicate outlier values of > 2.68 or < -2.68.
